# Genetic parameters for somatic cell score according to udder infection status in Valle del Belice dairy sheep and impact of imperfect diagnosis of infection

**DOI:** 10.1186/1297-9686-42-30

**Published:** 2010-07-26

**Authors:** Valentina Riggio, Baldassare Portolano, Henk Bovenhuis, Stephen C Bishop

**Affiliations:** 1 Dipartimento S.En.Fi.Mi.Zo.-Sezione Produzioni Animali, Università degli Studi di Palermo, Viale delle Scienze-Parco d'Orleans, 90128 Palermo, Italy; 2 Animal Breeding and Genomics Centre, Wageningen University, PO Box 338, 6700 AH Wageningen, The Netherlands; 3 The Roslin Institute and Royal (Dick) School of Veterinary Studies, University of Edinburgh, Roslin BioCentre, Midlothian EH25 9PS, UK

## Abstract

**Background:**

Somatic cell score (SCS) has been promoted as a selection criterion to improve mastitis resistance. However, SCS from healthy and infected animals may be considered as separate traits. Moreover, imperfect sensitivity and specificity could influence animals' classification and impact on estimated variance components. This study was aimed at: (1) estimating the heritability of bacteria negative SCS, bacteria positive SCS, and infection status, (2) estimating phenotypic and genetic correlations between bacteria negative and bacteria positive SCS, and the genetic correlation between bacteria negative SCS and infection status, and (3) evaluating the impact of imperfect diagnosis of infection on variance component estimates.

**Methods:**

Data on SCS and udder infection status for 1,120 ewes were collected from four Valle del Belice flocks. The pedigree file included 1,603 animals. The SCS dataset was split according to whether animals were infected or not at the time of sampling. A repeatability test-day animal model was used to estimate genetic parameters for SCS traits and the heritability of infection status. The genetic correlation between bacteria negative SCS and infection status was estimated using an MCMC threshold model, implemented by Gibbs Sampling.

**Results:**

The heritability was 0.10 for bacteria negative SCS, 0.03 for bacteria positive SCS, and 0.09 for infection status, on the liability scale. The genetic correlation between bacteria negative and bacteria positive SCS was 0.62, suggesting that they may be genetically different traits. The genetic correlation between bacteria negative SCS and infection status was 0.51. We demonstrate that imperfect diagnosis of infection leads to underestimation of differences between bacteria negative and bacteria positive SCS, and we derive formulae to predict impacts on estimated genetic parameters.

**Conclusions:**

The results suggest that bacteria negative and bacteria positive SCS are genetically different traits. A positive genetic correlation between bacteria negative SCS and liability to infection was found, suggesting that the approach of selecting animals for decreased SCS should help to reduce mastitis prevalence. However, the results show that imperfect diagnosis of infection has an impact on estimated genetic parameters, which may reduce the efficiency of selection strategies aiming at distinguishing between bacteria negative and bacteria positive SCS.

## Background

Somatic cell count (SCC), and therefore somatic cell score (SCS) have been widely promoted as an indirect method of predicting mammary infections [1] and as a selection criterion to improve mastitis resistance [2]. It has been demonstrated that mastitis is associated with an increase in SCC in small ruminants [3,4] and cattle [5,6]. Hence, milk with an elevated SCC is usually considered an indication of the occurrence of infection in the udder; and selection for decreased SCC could lead to reduced susceptibility to mastitis [7].

However, one difficulty in using SCC to find animals most resistant to mastitis is that factors known to influence SCC have different magnitude in healthy and infected animals [8], and SCC in healthy and in infected animals may even be considered as different traits. Indeed, it has been shown that cells in the milk from a healthy udder are mainly mammary gland epithelium and drain canal cells; whereas polymorphonuclear leukocytes (PMN) are the major cell population during early inflammation, playing a protective role against infectious diseases in the mammary gland [9,10]. Therefore, in principle SCC from healthy and infected animals should be analyzed separately. However, because the intramammary infection status is generally unknown, one model is usually applied indifferently to SCC obtained from all animals, irrespective of whether they are infected or not. Test-day SCC may, therefore, be regarded as a mixture of observations from animals with unknown health status [1]. We are in the fortunate position of having a dataset of SCC in dairy sheep for which bacteriological data are also available, indicating whether an animal was infected at the time of sampling. Therefore, instead of using mixture models to determine the infection status [1,11], we were able to analyze SCC, and therefore SCS, separately in apparently healthy and infected animals.

Fundamental to any diagnostic test are the concepts of sensitivity and specificity. Sensitivity (Se) measures the proportion of actual positives (i.e. diseased animals) which are correctly identified as such by the diagnostic test; whereas specificity (Sp) measures the proportion of negatives (i.e. healthy animals) which are correctly identified by the diagnostic test. If the diagnostic test is perfect, both *Se *and *Sp *are equal to unity. However, if the diagnostic test is imperfect, i.e. *Se *and *Sp *are less than unity, *Se *and *Sp *will influence classification of animals and potentially impact on estimable variance components and inferences drawn from the data. *Se *and *Sp *for the bacteriological assessments are unknown in our dataset, but it is likely that they were less than unity due to intermittent shedding of bacteria after infection and the possibility of contamination during sampling.

The aims of this study, therefore, were: (1) to estimate the heritability of SCS, according to whether the animals were healthy or infected, as assessed by our bacteriological data, along with the heritability of the infection status; (2) to estimate the phenotypic and genetic correlations between the bacteria negative SCS (i.e. apparently healthy animals) and the bacteria positive SCS (i.e. infected animals), and the genetic correlation between the bacteria negative SCS and the infection status; and (3) to evaluate the impact of imperfect diagnostic *Se *and *Sp *on variance component estimates for the traits of interest.

## Methods

### Dataset

The data consisted of 9,306 test-day records from 2,058 lactations of 1,125 ewes. Data for SCC were collected at approximately 1-month intervals, following an A4 recording scheme [12], by the University of Palermo in four Valle del Belice flocks between 2004 and 2007. At the same time, milk samples were collected aseptically from each animal for bacteriological analyses, which were performed by conventional techniques, on 5% sheep blood agar plates, incubated at 37°C, and examined after 10-24 h and 36-48 h incubation. The bacteriological colonies observed were mainly: *Staphylococcus aureus*, coagulase negative staphylococci, *Staphylococcus intermedius *and other staphylococci; *Streptococcus canis*, *Streptococcus dysgalactiae*, *Streptococcus uberis*, *Streptococcus agalactiae *and other streptococci; *Corynebacterium *spp., *Pasteurella *spp., and *Pseudomonas *spp. (Table 1). Ewes were considered infected if more than five colony forming units (CFU) per 10 μl of milk of one species of bacteria were isolated, and the data used in this study were the apparent presence or absence of infection for each milk sample.

**Table 1:** Number of observations and frequencies for bacteria observed

	Number of observations	Frequency (%)
*Staphylococcus aureus*	300	10.47
coagulase negative staphylococci	2316	80.81
*Staphylococcus intermedius*	36	1.26
Other staphylococci	20	0.70
*Streptococcus canis*	6	0.21
*Streptococcus dysgalactiae*	23	0.80
*Streptococcus uberis*	12	0.42
*Streptococcus agalactiae*	12	0.42
Other streptococci	84	2.93
*Corynebacterium *spp.	7	0.24
*Pasteurella *spp.	40	1.40
*Pseudomonas *spp.	10	0.34

All test-day records used in the analysis were required to have information regarding SCC and bacteriological status. After editing, the data comprised 8,843 test-day records from 2,047 lactations of 1,120 ewes. The pedigree file included 1,603 animals. In addition to the 1,120 animals with records, 84 sires and 399 dams without phenotypes were included in the pedigree. On average, the sires served at least two of the four flocks under study and they had 13.33 daughters on average.

For analyses investigating the properties of SCC in ewes with either positive or negative bacteriological status, we divided the data in two sub-datasets: one sub-dataset comprising test-day records with the presence of infection (bacteria positive) and the accompanying SCC information (2,866 test-day records from 1,263 lactations of 805 ewes), and the other one comprising test-day records with the absence of infection (bacteria negative) and the accompanying SCC information (5,977 test-day records from 1,805 lactations of 1,062 ewes). Because the dataset was divided by test-day records, the same animals could appear in both sub-datasets and they could even appear in both datasets in the same lactation. Of the 1,120 ewes from the original data, 744 were included in both sub-datasets.

### Statistical Analyses

The test-day traits analyzed as response variables were SCS and the infection status. SCS were obtained after log-transformation of test-day SCC, using a base 2 logarithmic function: SCS = log_2 _(SCC/100) + 3 [13], in order to get an approximated normal distribution for this trait. An infection status trait was created, based on the presence/absence of pathogens, indicating whether ewes were infected (1) or apparently healthy (0) at each test-day.

Variance components and genetic parameters for SCS (whole dataset as well as bacteria negative and positive subsets) were estimated using ASReml [14]. The following repeatability test-day animal model as described by Riggio et al. [15] was used to analyze the data:

where *y*_*ijklmn *_was the SCS test-day measurement; *μ *was the population mean; *FTD*_*i *_was the random effect of flock by test-day interaction *i *(91 levels); *YPS*_*j *_was the fixed effect of year by season of lambing interaction *j *(6 levels), where the season of lambing was coded as 1 if a ewe gave birth in the period January through June, otherwise it was coded as 2 [15]; *P*_*k *_was the fixed effect of the parity (3 levels); *LS*_*l *_was the fixed effect of litter size class *l *(2 levels, single or multiple born lambs); *DIM*_*ijklmn *_and exp(-0.05* *DIM*_*ijklmn*_) were two covariates used to model the shape of lactation curves [16]; *A*_*m *_was the random additive genetic effect of the individual *m *(1,603 levels); *PE*_*m *_was the general random permanent environmental effect of ewe *m *across lactations (1,120 levels); *PE*_*km *_was the random permanent environmental effect on the individual *m *within parity class *k *(2,047 levels); *e*_*ijklmn *_was the random residual effect. The same model was used for the analysis of the two sub-datasets.

Variance components and heritability for the infection status were first estimated using an animal linear model accounting for the same effects included in the model used for SCS. Then, a threshold animal model was fitted, assuming a probit link function.

Phenotypic and genetic correlations between SCS in the bacteria negative and positive subsets were estimated using bivariate analyses, fitting the same fixed and random effects as previously described. Given the data structure, i.e. non-contemporaneous bacteria negative and positive SCS observations for any individual, the environmental covariance between the two traits was assumed to be zero and not estimated when the genetic correlation was estimated. However, covariances were fitted for the additive genetic term and for the permanent environmental effects of the ewe both across and within lactations. To estimate an approximated phenotypic correlation, the data were restructured and reduced to adjacent pairs of bacteria negative and positive SCS data, i.e. the bacteria negative and positive SCS observations closest within one lactation were used. It should be noted that this approach does create a unique subset of SCS samples, as the bacteria negative SCS samples are from ewes either immediately prior to or post infection; conversely the bacteria positive SCS sample are from recovering or newly infected ewes. The same fixed effects, as previously described, were fitted but the random effects model was simplified with (co)variance terms estimated only for additive genetic and residual effects.

The genetic correlation between the bacteria negative SCS and the infection status was estimated using TM (Threshold Model) program (available upon request to the author andres.legarra@toulouse.inra.fr), using a Bayesian analysis and performing numerical integration through the Gibbs sampler. The TM program does not handle covariates, so in this case the model was simplified and the two covariates of DIM were excluded. Flat priors were used both for fixed effects and variance components. A chain of 100,000 iterations was used, discarding the first 30,000 samples and saving a sample every 10 iterations. The mean of the estimated marginal posterior density has been used as point estimate of the genetic parameters of interest.

Genetic parameters for infection status, bacteria negative SCS, and bacteria positive SCS are potentially affected by imperfect *Sp *and *Se*, which were both implicitly assumed to be unity in the variance component estimation analyses. Additional file 1 shows the principles of the calculations used to show how imperfect *Se *and *Sp *can influence the interpretations of these data. Using the observed variance components, likely impacts of imperfect *Sp *and *Se *on estimated mastitis prevalence, predicted differences between SCS in bacteria negative and positive animals, and variance components were explored.

## Results

**Figure 1:**
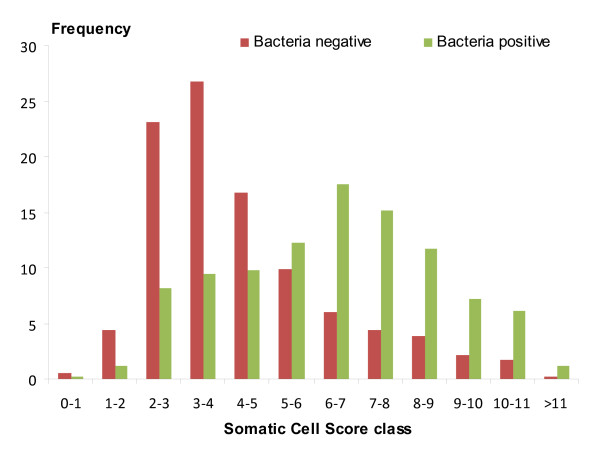
**Distribution of bacteria negative and bacteria positive SCS**. Distribution of bacteria negative (i.e. healthy) and bacteria positive (i.e. infected) SCS for the observed prevalence of bacteria positive milk samples (p' = 0.32).

Arithmetic means, standard deviations and range of SCC and SCS test-day traits are given in Table 2. The geometric mean SCC was 403 (× 10^3 ^cells/mL) for the whole data, 253 for the bacteria negative, and 1,082 for the bacteria positive. Although ranges of SCC for uninfected and infected animals were similar, the arithmetic mean SCC for infected animals was approximately 3-fold higher than that for uninfected animals. This result suggests that although the distributions of bacteria negative and bacteria positive SCS partially overlap, they are substantially different as shown in Figure 1. The difference between bacteria positive and bacteria negative SCC may have been higher if SCC and infection status had been considered per udder half. However, we had only information at the animal level (summarizing the whole udder); therefore a dilution effect due to the mixing of milk with high SCC coming from infected glands and milk with low SCC from a healthy gland has to be considered.

**Table 2:** Descriptive statistics of SCC and SCS traits

	Mean	SD	Range
Whole data SCC (× 10^3 ^cells/ml)	1,812	4,150	13 - 31,268
Whole data SCS	5.01	2.37	0.06 - 11.29

Bacteria negative SCC (× 10^3 ^cells/ml)	1,077	3,084	13 - 29,368
Bacteria negative SCS	4.34	2.06	0.06 - 11.20

Bacteria positive SCC (× 10^3 ^cells/ml)	3,346	5,462	16 - 31,268
Bacteria positive SCS	6.42	2.36	0.36 - 11.29

Phenotypic, genetic, and environmental variances after adjustment for fixed effects, heritabilities and repeatabilities within and across lactations for SCS traits are given in Table 3. The heritability estimate for SCS was 0.09. However, estimates for bacteria negative and bacteria positive SCS were respectively 0.10 and 0.03. This difference could be due in part to the different sub-datasets (i.e. different animals and different number of records) used for the analysis. Therefore, an analysis was carried out in which only the animals present in both sub-datasets were considered. However, this had little effect on the estimated heritabilities and did not change the interpretation of the results. The observed phenotypic variance was 5.57 for infected animals and 2.23 for bacteria negative animals; whereas the observed genetic variance was 0.16 for infected animals and 0.22 for bacteria-negative animals. Repeatability estimates within lactations ranged between 0.20 and 0.29, whereas repeatability estimates across lactations ranged between 0.30 and 0.33, and were higher than the within lactation values.

**Table 3:** Genetic parameters* for SCS traits

	**σ**^**2**^_**p**_	**σ**^**2**^_**a**_	**σ**^**2**^_**e**_	**h**^**2 **^**± SE**	**r**_**wit **_**± SE**	**r**_**acr **_**± SE**
Whole data SCS	5.467	0.492	2.633	0.09 ± 0.04	0.29 ± 0.04	0.33 ± 0.02
Bacteria negative SCS	2.225	0.223	1.188	0.10 ± 0.06	0.21 ± 0.04	0.30 ± 0.03
Bacteria positive SCS	5.573	0.161	2.554	0.03 ± 0.03	0.20 ± 0.05	0.31 ± 0.04

*Phenotypic (σ^2^_p_), genetic (σ^2^_a_), and environmental (σ^2^_e_) variances, heritability (h^2^) and repeatability within (r_wit_) and across (r_acr_) lactations (± SE) for SCS traits

Table 4 shows the heritabilities of the infection status, estimated by considering the infection status both as a binary and continuous trait on the underlying scale, i.e. liability to infection, and the expected value on the underlying scale calculated from the binary scale using the approximation of Dempster and Lerner [17]. The heritability estimate obtained with the probit model was 0.09. As expected, the heritability estimate from the normal analysis was somewhat lower, and it can be seen that the assumption of the trait being continuous with normally distributed residuals is violated. However, the expected value on the underlying scale derived from the heritability estimate obtained with the normal analysis was the same as that from the binary trait analysis, confirming that the impact of departures from normality is predictable.

**Table 4:** Heritability for infection status with normal and probit analysis

	Normal analysis* h^**2 **^**± SE**	Probit analysis** h^**2 **^**± SE**	**Expected value**^**†**^ **h**^**2**^
Infection status	0.05 ± 0.02	0.09 ± 0.04	0.09

*Treating the infection status as a continuous variable.

**Treating the infection status as a binary trait.

^†^Calculated with Dempster and Lerner's formula [17].

The phenotypic and genetic correlations between bacteria negative and bacteria positive SCS, and the genetic correlation between bacteria negative SCS and the infection status are presented in Table 5. The phenotypic correlation between bacteria negative and bacteria positive SCS was 0.19 (s.e. 0.02); whereas the genetic correlation was 0.62 (s.e. 0.12), indicating that whilst there is a moderate positive correlation between these traits it may be more appropriate to consider them as different traits. The genetic correlation between bacteria negative SCS and the infection status was 0.51, suggesting that animals with lower SCS, assessed when apparently not infected, are genetically less likely to be infected (across all time points). For completeness we also estimated the genetic correlation between SCS in bacteria positive animals and liability to infection. The estimated correlation was 0.81 but its biological interpretation is not obvious to us.

**Table 5:** Correlations* between SCS and infection status

		**Bacteria positive SCS**	**Infection status**
	
Bacteria negative SCS	Genetic correlation:	0.62 ± 0.02	0.51
	Phenotypic correlation:	0.19 ± 0.02	***

*Genetic and phenotypic correlations (± SE**) between bacteria negative SCS and bacteria positive SCS, and genetic correlation between bacteria negative SCS and infection status

**SE is not reported for the correlation between bacteria negative SCS and infection status, as it was estimated using a Bayesian approach

***No attempt was made to estimate a phenotypic correlation between bacteria negative SCS and infection status

**Figure 2:**
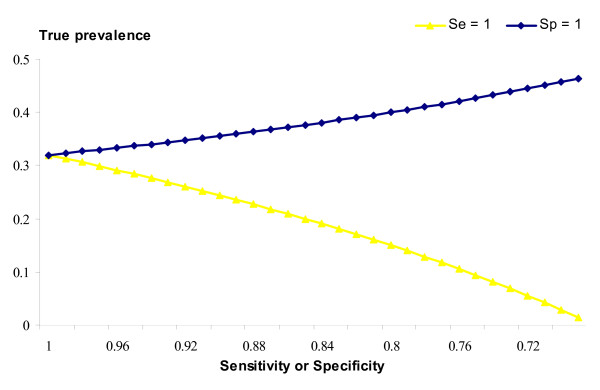
**True prevalence depending on imperfect specificity and sensitivity**. Trend of the true prevalence of infection depending on imperfect specificity (*Se *= 1) or imperfect sensitivity (*Sp *= 1) for the observed prevalence of bacteria positive milk samples (p' = 0.32).

**Figure 3:**
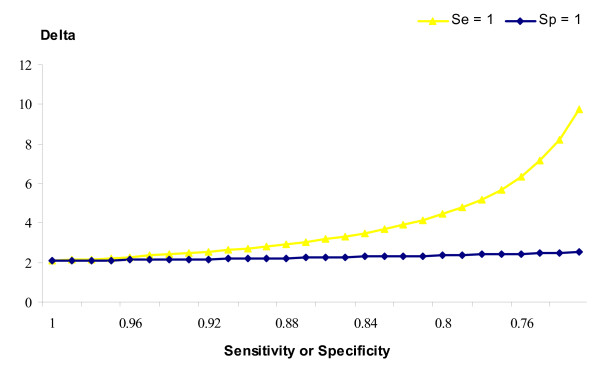
**True difference between healthy and infected SCS**. Trend of the true difference (Delta) between SCS in healthy and infected populations depending on imperfect specificity (*Se *= 1) or imperfect sensitivity (*Sp *= 1).

All analyses so far were done assuming the *Sp *= *Se *= 1. This may not be the case; although we have no data on the accuracy of the diagnoses, they are unlikely to be perfect. The impacts of imperfect diagnoses can be tabulated from formulae derived in Additional file 1. The impact of imperfect *Sp *or *Se *on the true prevalence, given the observed prevalence, is shown in Figure 2. If the *Se *is less than unity, then the true prevalence will have been underestimated, whereas if *Sp *is less than perfect then the true prevalence will have been overestimated. Not only does the true prevalence of infection changes as *Sp *or *Se *change, but the estimated true difference in SCS between healthy and infected animals also changes, as shown in Figure 3. Less than perfect *Se *has little impact on the true difference between healthy and infected animals, whereas if *Sp *is less than perfect then the true difference between healthy and infected animals will have been underestimated. Moreover, once *Sp *drops below ~ 0.8 the estimated differences between the two populations becomes improbably large.

**Figure 4:**
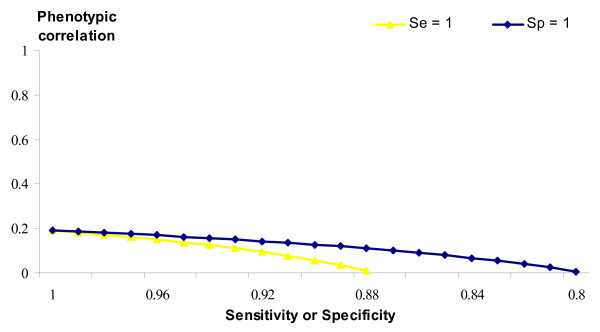
**True phenotypic correlation between healthy and infected SCS**. Trend of the true phenotypic correlation between SCS in healthy and infected populations depending on imperfect specificity (*Se *= 1) or imperfect sensitivity (*Sp *= 1).

**Figure 5:**
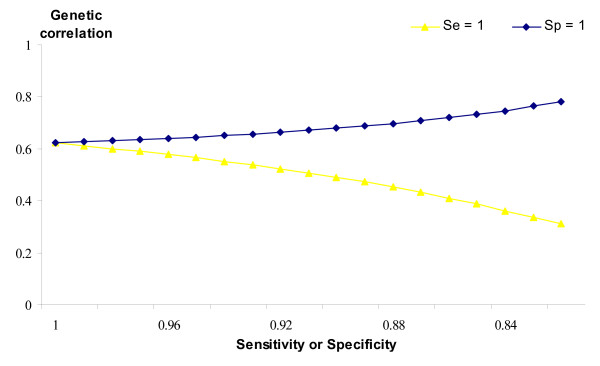
**True genetic correlation between healthy and infected SCS**. Trend of the true genetic correlation between SCS in healthy and infected populations depending on imperfect specificity (*Se *= 1) or imperfect sensitivity (*Sp *= 1).

Phenotypic and genetic correlations between SCS in infected and healthy populations also change as *Sp *or *Se *change, as shown in Figures 4 and 5. If both *Se *and *Sp *are less than unity, the true phenotypic correlation will have been slightly underestimated. However, imperfect *Sp *has a larger effect, as the true phenotypic correlation drops more rapidly. A different trend is reported for the true genetic correlation (Figure 5), which will have been underestimated, if *Sp *is less than unity; whereas if *Se *is less than perfect then true genetic correlation will have been overestimated. Although *Sp *and *Se *are unknown in these data, the improbable expected results when either or both values are low suggest that both parameters are likely to be somewhat higher than 0.8.

## Discussion

This paper demonstrates that SCC, and therefore SCS, of apparently uninfected and infected animals are most likely two different traits with different heritabilities. We have shown that bacteria negative SCS has a slightly higher heritability than the infection status (i.e. likely mastitis) and that bacteria negative SCS (i.e. from apparently uninfected animals) is positively genetically correlated with both bacteria positive SCS (i.e. from infected animals) and infection status. Finally, we have explored the implications of less than perfect *Se *and *Sp *on our estimates. Possibly the greatest impact of less than perfect diagnosis is on the heritability of liability to mastitis, which is likely to be somewhat underestimated if the diagnostic test is poor. This is likely to decrease potential genetic progress for improved resistance.

Evidence has been published that healthy ewes normally have higher SCC than healthy cows [18-20]. Bufano et al. [21] have shown that high SCC (> 1 million/mL) do occur in healthy sheep's milk, especially towards the end of lactation. Therefore, whereas in cattle SCC is widely recognized as indicator of mastitis, results on the efficiency of SCC as an indicator trait are inconsistent in dairy sheep studies. However, Ariznabarreta et al. [22] and Gonzalo et al. [2] have demonstrated that for around 70% of mammary pathogens isolated from ewes with subclinical mastitis, their presence in ewe milk is associated with high SCC. Therefore, published evidence exists that mastitis does accompany an increase in SCC in sheep [23]. Moreover, Leitner et al. [24] have suggested that because sheep have only two mammary glands, dilution effects due to the mixing of milk with high SCC from an infected gland, and milk with low SCC from a healthy gland, will be relatively small at the animal level. Besides, in dairy cows, subclinical mastitis, with a frequency ranging from 20-50% [10,25] may be less apparent because the increase in SCC in an infected gland is modest (about 300-500 × 10^3 ^cells/mL) and the mixing with the milk from uninfected quarters is sufficient in most cases to appreciably lower the effect of SCC at the cow level [26].

The mean SCS for bacteria negative animals was similar to the value of 4.86 reported by Ariznabarreta et al. [22] and 5.15 reported by Leitner et al. [23]; whereas the mean SCS for infected animal was similar to the value of 6.32 reported by Leitner et al. [23] in Israeli-Assaf and Awassi sheep. The observed difference between the bacteria positive and negative populations was 2.08, i.e. suggesting a four-fold difference in SCC between typical diseased and healthy individuals. However, if only one half of the udder was infected, then due to the dilution this would equate to an eight-fold difference between healthy and infected halves, assuming independence (i.e. infection in one half, which results in an increase in SCC, does not increase SCC in the other half). If *Se *was in fact less than perfect, this would only have slightly influenced the true difference (delta) between the two populations; whereas if *Sp *was less than perfect (i.e. healthy animals wrongly classified as being infected) then the difference between the two populations would have been considerably underestimated.

The heritability estimates for overall SCS and SCS in apparently healthy animals were generally in the range reported in the literature for repeatability test-day models i.e. 0.04 to 0.16 [15,27,28]. Other studies have reported higher heritability estimates for the average SCS during lactation, from 0.11 to 0.18 [29-31]. However, the heritability for SCS in infected ewes (0.03) was at the low end of published values. It is important to highlight that the similarity between the heritability for bacteria negative SCS and that usually observed for SCS is probably due to the fact that the former refers to a mix of repeatable healthy animals, animals that have recovered from infection, and infected animals with incorrect diagnosis. On the contrary, SCS in infected animals are mostly truly positive samples, and the low heritability actually reflects that most of the variation in these samples is non-genetic. The high environmental variance for the bacteria positive SCS is possibly due to the nature of the pathogens (i.e. hosts may respond differently to infection by a pathogen or another) and the sinusoidal variation of SCC after infection, both of which would increase variation in the dataset.

Estimated repeatabilities were similar for the two sub-datasets. Repeatability estimates within lactations ranged between 0.20 and 0.29, and were in the range reported in the literature for sheep i.e. 0.22 to 0.38 [28,32,33]. However, repeatability estimates across lactations ranged between 0.30 and 0.33, and were higher than the value of 0.13 reported by Serrano et al. [33] for the Manchega breed.

The estimated genetic correlation between bacteria negative and bacteria positive SCS (0.62) is positive and moderate, but significantly less than unity. Therefore, our results suggest that bacteria negative and bacteria positive SCS may be partially independent traits, possibly with different heritabilities. It might be hypothesized that ewes with high bacteria negative SCS also have a higher reaction, in terms of increase in SCS, in response to an infection. It has to be taken into account that the genetic correlation might partially reflect the fact that the dataset of bacteria negative SCS animals also includes previously infected animals. However, a somewhat different interpretation is possible. The bacteria positive SCS actually consists of the bacteria negative SCS (i.e. the SCS ewes would have had in the absence of infection) along with the true response to infection. Therefore, it is likely that the positive genetic correlation is picking up the baseline that is contributing to both measures, with the true response (i.e. the extra) SCS possibly being uncorrelated. The sum of the two results in a trait that is genetically correlated with bacteria negative SCS, but has a low phenotypic correlation (0.19). The exploration of sensitivity and specificity suggests that imperfect diagnosis of the infection has only minor impacts on the correlation, with the impacts becoming large only when the diagnostic tests are very poor.

Very few data on intramammary infection assessed by bacteriological analyses are found in the literature, and published studies refer more directly and exhaustively to udder health status. In cattle, heritabilities for intramammary infection varied from 0.02 to 0.04 as reported by Weller et al. [34], and were somewhat higher (0.10 to 0.20) in Detilleux et al. [35] and Wanner et al. [36]. Our value of 0.09 falls into the mid range of published values. However, an important result we found was that with imperfect *Se *and, particularly, *Sp*, the heritability of liability is likely to be substantially underestimated. In other words, there may truly be more genetic variation for liability to mastitis than the field data suggest. No estimates, however, are reported for the genetic correlation between bacteria negative SCS and the infection status. Our results, perhaps surprisingly, suggest a positive genetic correlation between bacteria negative SCS and liability, suggesting that animals with higher bacteria negative SCS are more liable to have mastitis. This is a result that requires independent validation but it does suggest that the approach of selecting animals for decreased SCS, even in the absence of knowledge about infection status, is correct and will help to reduce the prevalence of mastitis.

The choice of diagnosis criteria is important, as it affects the probability that healthy animals are truly diagnosed as healthy and that infected animals are classified as such. Therefore, as our results have shown, biases may be quite large when diagnostic criteria are not sensitive or specific enough. Our results show that the imperfect diagnosis of infection has an impact on estimated genetic parameters, particularly the heritability of liability, and some of the inferences drawn from the data. Bacteriological examination is often considered to be the 'golden standard' for routine detection and identification of major mastitis pathogens, and is usually assumed to be perfect, i.e. *Sp *= *Se *= 1. However, even good quality bacteriological or clinical mastitis data will most likely have true *Se *and *Sp *values somewhat less than one. Some cases will be missed, others may be mis-diagnosed. Hence, the answers we get may not be quite what we think they are, and we may well be underestimating the impacts of mastitis and the potential for selecting animals for enhanced resistance.

## Conclusions

Our results suggest that bacteria negative and bacteria positive SCS may be partially independent traits, confirming that SCC from healthy and infected animals should be analyzed separately. Moreover, a positive genetic correlation between bacteria negative SCS and liability to mastitis was found, suggesting that the approach of selecting animals for decreased SCS will help to reduce the prevalence of mastitis. However, our results show that the imperfect diagnosis of infection has an impact on estimated genetic parameters. Hence, the impacts of mastitis and the potential for selecting animals for enhanced resistance may well be underestimated.

## Competing interests

The authors declare that they have no competing interests.

## Authors' contributions

VR conceived and designed the study, contributed to the sampling, elaborated data, and drafted the manuscript. BP contributed to the sampling and data elaboration, supervised the work, and funded the study. HB supervised the work and was involved in the design of the study. SCB supervised the work, elaborated data, and was involved in drafting the manuscript and in the design of the study. BP, HB, and SCB revised critically the manuscript and data. All authors reviewed the manuscript and accepted the final version.

## Supplementary Material

Additional file 1 **Effect of imperfect sensitivity and specificity on means and variances of continuous traits**. The word file provided shows the principles of the calculations used to show how imperfect sensitivity and specificity can influence animals' classification and impact on estimated variance components. Click here for file
