# Supplementary material for: Genetic parameters for somatic cell score according to udder infection status in Valle del Belice dairy sheep and impact of imperfect diagnosis of infection
Source: Genet Sel Evol. 2010 Jul 26;42(1):30. doi: 10.1186/1297-9686-42-30 (PMC2915952; doi:10.1186/1297-9686-42-30)
Supplement: Additional file 1 — Effect of imperfect sensitivity and specificity on means and variances of continuous traits. The word file provided shows the principles of the calculations used to show how imperfect sensitivity and specificity can influence animals' classification and impact on estimated variance components. [file 1297-9686-42-30-S1.DOC]

**Additional file 1 - Effect of imperfect sensitivity and specificity on means and variances of continuous traits**

Proportions of animals classified as healthy or diseased, as a function of specificity () or sensitivity (), where *p* and *p’* are the true and observed prevalence, are:

|  | | Test Classification | |  |
| --- | --- | --- | --- | --- |
|  |  | healthy | diseased | Sum |
| Disease | healthy |  |  |  |
|  | diseased |  |  |  |
|  | Sum |  |  |  |

1) The true prevalence is

2) Let H and H’ signify true and observed healthy animals, and D and D’ signify true and observed diseased animals. The means for the observed healthy and diseased populations can be considered as weighted averages of the true population means:

and

where:$X_{1}=\frac{\left(1-p\right)Sp}{1-p'}$,$X_{2}=\frac{p\left(1-Se\right)}{1-p'}$, $X_{3}=\frac{\left(1-p\right)\left(1-Sp\right)}{p'}$, and $X_{4}=\frac{pSe}{p'}$.

Therefore, the true difference between diseased and healthy animals, after noting that $X_{1}=1-X_{2}$and $X_{3}=1-X_{4}$, and after simplification, is given by:

$$\Delta=\left(\mu_{D'}-\mu_{H'}\right)\left\{\left(S_{p}+S_{e}-1\right)p\left(1-p\right)/\left[p'\left(1-p'\right)\right]\right\}^{-1}$$

Solving for *p*, this formula may be rewritten:

$$\Delta=\left(\mu_{D'}-\mu_{H'}\right)p'\left(1-p'\right)\left(S_{p}+S_{e}-1\right)/\left[\left(p'+S_{p}-1\right)\left(S_{e}-p'\right)\right]$$

3) The variance of and are variances of distributions comprising different proportions healthy and diseased animals. In general, a mixture of 2 distributions Y and Z, in proportions *p* and *(1-p)* will have a variance:

.

Therefore, the true variances for healthy and diseased animals can be deduced from the following equations:

and

$$V\left(D'\right)=X_{3}\sigma_{H}^{2}+X_{4}\sigma_{D}^{2}+X_{3}X_{4}\Delta^{2}$$

4) For the covariance of and , comparing the same individual, we have 4 possibilities:

| Classification | |  |
| --- | --- | --- |
| Healthy | Diseased | Frequency |
| True healthy | True healthy |  |
| True healthy | True diseased |  |
| True diseased | True healthy |  |
| True diseased | True diseased |  |

The covariance is therefore:

From this equation it is possible to deduce the true covariance between healthy and diseased animals, and the implied true correlation can be constructed from the estimated true variances and true covariance terms.
